# Effects of habitat structure and land-use intensity on the genetic structure of the grasshopper species *Chorthippus parallelus*

**DOI:** 10.1098/rsos.140133

**Published:** 2014-10-22

**Authors:** Kerstin R. Wiesner, Jan Christian Habel, Martin M. Gossner, Hugh D. Loxdale, Günter Köhler, Anja R. R. Schneider, Ralph Tiedemann, Wolfgang W. Weisser

**Affiliations:** 1 Institute of Ecology, Friedrich-Schiller-University, 07742 Jena, Germany; 2 Terrestrial Ecology Research Group, Department of Ecology and Ecosystem Management, School of Life Sciences Weihenstephan, Technische Universität München, 85354 Freising, Germany; 3 Unit of Evolutionary Biology/Systematic Zoology, Institute of Biochemistry and Biology, University of Potsdam, 14476 Potsdam, Germany

**Keywords:** agricultural intensification, edge effects, genetic diversity, genetic differentiation, land-use index

## Abstract

Land-use intensity (LUI) is assumed to impact the genetic structure of organisms. While effects of landscape structure on the genetics of local populations have frequently been analysed, potential effects of variation in LUI on the genetic diversity of local populations have mostly been neglected. In this study, we used six polymorphic microsatellites to analyse the genetic effects of variation in land use in the highly abundant grasshopper *Chorthippus parallelus*. We sampled a total of 610 individuals at 22 heterogeneous grassland sites in the Hainich-Dün region of Central Germany. For each of these grassland sites we assessed habitat size, LUI (combined index of mowing, grazing and fertilization), and the proportion of grassland adjoining the sampling site and the landscape heterogeneity (the latter two factors within a 500 m buffer zone surrounding each focal site). We found only marginal genetic differentiation among all local populations and no correlation between geographical and genetic distance. Habitat size, LUI and landscape characteristics had only weak effects on most of the parameters of genetic diversity of *C. parallelus*; only expected heterozygosity and the grasshopper abundances were affected by interacting effects of LUI, habitat size and landscape characteristics. The lack of any strong relationships between LUI, abundance and the genetic structure might be due to large local populations of the species in the landscape, counteracting local differentiation and potential genetic drift effects.

## 2. Introduction

Agricultural intensification has led to a decrease in the quality of many semi-natural habitats. Biota living in the remaining habitat islands often suffers from increasing fragmentation and thus a rise of geographical isolation accompanied by a decrease in habitat size and potential negative edge effects [1]. Negative edge effects can be observed if the surrounding landscape gets intensively used and, vice versa, positive edge effects occur if the population gets surrounded by landscape structures that provide suitable habitat structures. Especially populations in small and isolated habitat remnants can be strongly affected by environmental and demographic stochasticity and may show strong population-size fluctuations [2]. Consequently, such populations are more often subjected to population bottlenecks and are at a much higher risk of local extinction than large and interconnected populations [1]. In addition, genetic drift and subsequent loss of genetic diversity further endanger these populations [3]. For a number of invertebrates, in particular more specialized species, lower levels of genetic diversity have been found [4]. The surrounding landscape and local land-use intensity (LUI) may also affect genetic diversity [1].

Apart from these various drivers related to landscape and habitat structures, further characteristics such as species' distribution, ecology (e.g. ecological amplitude of a species) and behaviour (e.g. dispersal ability) play a pivotal role. Most studies in population genetics highlight the first criteria (landscape and habitat structures) as being crucial [5], but only little is known about the effects of habitat quality on the abundance of species [6] and subsequently on the genetic diversity [7]. For example, studies on the common grasshopper *Chorthippus parallelus* showed that individual fitness strongly depends on dietary mixing and thus on the composition of the vegetation. Survival as well as fecundity of *C. parallelus* increased with the number of foodplants in experimental studies [8,9]. Dietary mixing has thus been proposed as a possibility for generalists to overcome nutritional deficiencies of single plant species. Other previous mesocosm field experiments showed that plant functional group diversity (but not plant species richness *per se*) affects the performance and fitness of this grasshopper species significantly [7,10].

Most population genetic studies focus on specialized species [5]. However, rather little is known for widespread and common species. In this study, we analyse the effects of habitat structures on the population genetic structure of the widespread meadow grasshopper, *Chorthippus parallelus* (Zetterstedt 1821). This species is the most common grasshopper of Europe and can be found in most meadows, even if they are intensively used [11]. Samples were collected at 22 grassland sites located in the Hainich-Dün region in Central Germany. The study sites cover contrasting habitat characteristics and provide plots with different management regimes, ranging from highly intensive to extensive. For each site, we assessed the habitat size, the LUI and the environment such as the proportion of grasslands and the degree of habitat heterogeneity in a 500 m buffer around each sampling point. We sampled a total of 610 individuals of *C. parallelus* and analysed six polymorphic microsatellites. Based on these data, we calculated parameters of genetic differentiation and diversity and investigated the relationships of genetic diversity and land-use characteristics. In detail, we ask the following questions:

(i) Are populations of *C. parallelus* genetically differentiated across our study region?
(ii) Do habitat size, LUI and/or the surrounding environment affect the abundance and genetic diversity of *C. parallelus*?
(iii) Does local abundance affect the genetic diversity of *C. parallelus*?

## 3. Material and methods

### 3.1 Study region

**Figure 1.**
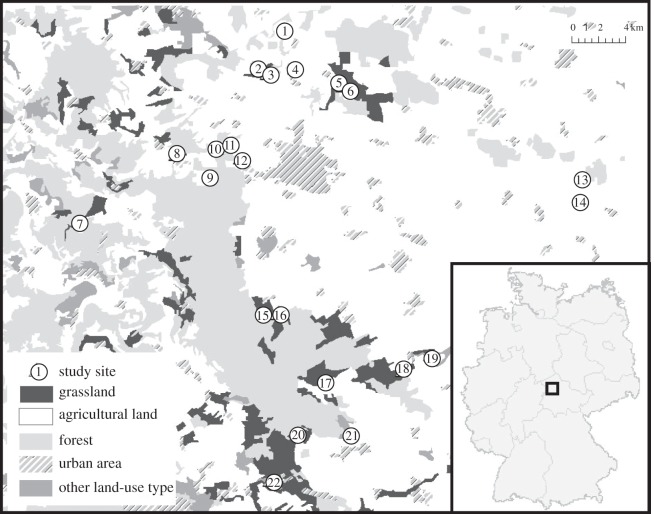
Location of the study region in Germany (small map), and overview of all study sites and land-use classifications in the Hainich-Dün region (large map). Given numbers coincide with all tables.

The study was conducted in the Hainich-Dün region in the northwest of Thuringia, Central Germany. This area is part of the German Biodiversity Exploratories Project [12]. The landscape is dominated by agriculture, including arable land and grasslands, but also contains one of the largest continuous broad-leaved forests in Germany. The proportion of grasslands is comparatively small as major parts comprise intensively used arable land (figure 1).

### 3.2 Study sites and land-use intensity

For each of the 22 grassland sampling localities, we assessed habitat size and various land-use types: hay meadows (six sites), mown pastures (eight sites) and pastures (eight sites). Meadows were mown once (one site) twice (four sites) or three-times (one site) per year. Hay meadows and mown pastures were fertilized. Mown pastures and pastures were grazed either by sheep (seven sites) or cattle (nine sites). For our analyses, we used a quantitative, continuous index of LUI. This index combines the main human management measures of the grasslands, mowing, grazing and fertilization, into a single standardized index (see [13] for a detailed description of how the index LUI is calculated). For this study, we used averaged management measures over the period 2006–2008. For statistical analyses, we additionally carried out analysis using land-use categories, by dividing all sites into the three categories: low LUI (index less than 1.5), medium LUI (between 1.5 and 2.0) and high LUI (more than 2.0).

To analyse the effect of landscape structure, we assessed the environment in a 500 m buffer around a grassland plot on the basis of CORINE (2006) data (Pan-European project CORINE land cover—CLC, available from http://www.corine.dfd.dlr.de, accessed January 2014). From this, we calculated the percentage of grassland (CORINE types 231, 321) in the circle, the most suitable habitat of *C. parallelus*. Additionally, we calculated habitat diversity as Shannon diversity index based on the surrounding environment, split into the following categories: arable land (CORINE types 211, 242, 243), forest (CORINE types 311, 312, 313, 324), urban land (CORINE types 111, 112, 121, 122, 123, 124, 131, 132, 133, 141, 142), woodland (CORINE types 221, 222) and water bodies (CORINE types 511, 512). Details on each sampling site and its environmental conditions are given in table 1.

**Table 1.** Overview of environmental conditions of each sampling site. Given are details on habitat size (ha) calculated for the year of sampling (2009), land-use practices (mowing, grazing and fertilization), the respective LUI averaged over the years 2006–2008, the categories of LUI (low, medium and high intensity), the percentage of grassland in the surrounding 500 m buffer, the environmental heterogeneity expressed by the Shannon diversity index calculated for the 500 m buffer, and the abundance of individuals based on sweep-net samples.

			land use					landscape (*r*=500 m)		abundance (2008–2012)	
site	site^*a*^	size (ha)	mowing	grazing	fertilization	LUI	LUI category	grassland (%)	Shannon div. index	total	mean
1	HEG43	12.51	no	yes	no	0.74	low	0	0.18	48	7.0
2	HEG07	65.02	no	yes	no	1.89	medium	77.8	0.66	6	3.0
3	HEG08	65.02	no	yes	no	1.89	medium	81.1	0.61	17	5.5
4	HEG10	2.63	yes	no	yes	1.15	low	0	0.63	22	9.5
5	HEG28	2.68	yes	no	yes	2.00	medium	88.0	0.37	19	7.0
6	HEG29	8.38	yes	no	yes	1.84	medium	67.5	0.66	18	5.0
7	HEG31	11.19	yes	yes	yes	2.01	high	13.9	0.99	40	9.0
8	HEG05	5.13	yes	yes	yes	2.40	high	52.5	1.12	3	0.5
9	HEG30	5.09	yes	no	yes	2.42	high	0	0.69	5	1.5
10	HEG41	128.36	no	yes	no	0.59	low	50.1	1.02	4	1.5
11	HEG09	21.00	no	yes	no	0.78	low	33.4	0.86	22	10.0
12	HEG06	22.64	yes	yes	yes	2.05	high	0	0.34	60	24.5
13	HEG46	2.88	no	yes	no	0.66	low	0	0.55	30	14.5
14	HEG21	12.20	no	yes	no	0.67	low	0	0.00	10	5.0
15	HEG33	14.57	yes	yes	yes	1.86	medium	71.4	0.65	5	1.0
16	HEG04	7.59	yes	yes	yes	1.96	medium	63.5	0.71	14	2.5
17	HEG15	10.26	yes	yes	yes	2.04	high	65.3	0.83	6	1.5
18	HEG32	21.66	yes	yes	yes	2.24	high	70.3	0.73	25	8.0
19	HEG27	5.20	yes	no	yes	1.86	medium	30.7	0.62	11	5.0
20	HEG16	18.00	no	yes	no	0.85	low	47.1	0.69	24	11.5
21	HEG37	7.91	yes	yes	yes	2.16	high	0	0.01	124	24.0
22	HEG02	19.83	yes	no	yes	2.73	high	55.2	0.69	8	1.0

^*a*^Name of site according to the Biodiversity Exploratories, also given in the tables of the electronic supplementary material.

### 3.3 Abundance of *Chorthippus parallelus*

We used standardized sweep-net samples (round sweep net with a 30 cm diameter) to assess the abundance of *C. parallelus*. Twenty double sweeps along each of three transects (total 60 double sweeps) were performed twice a year (June and August) during the years 2008–2012 by the same person. Arthropods collected from the three transects were pooled and preserved in 70% ethanol. Orthopterans were separated from other insects. Individuals of *C. parallelus* were counted. We used the summed number of individuals over the 5 years as measure of *C. parallelus* abundance to account for fluctuations in population densities among years. We neither performed further census calculations nor calculated any density estimates, as studies showed that extrapolations of sweep netting on square metres and meadow sizes will result in much more inaccurate values because of the unknown local species distribution [11,14].

### 3.4 Molecular analyses

Between 20 and 33 individuals of *C. parallelus* were collected during July 2009 (including juveniles and adults), with a mean of 26±3 individuals per site (table 1). Individuals were stored in 98% ethanol at 4^°^C until DNA extraction. Genomic DNA was extracted from postfemur muscles of the hind leg using a ‘salting out’ procedure [15]. Six previously designed polymorphic microsatellites were genotyped (Cpara_D5, Cpara_IIB-F9, Cpara_B-F1, Cpara_IIB-G5, Cpara_C-D6, Cpara_B-H5, see [16]) [17]. DNA amplification and fragment length detection was conducted as described previously [15,18].

### 3.5 Population genetic analyses

We tested for distortion of microsatellite data due to stutter bands, large allele dropout or null alleles using the program Micro-Checker v. 2.0 [19]. Tests of Hardy–Weinberg equilibrium and linkage disequilibrium were conducted with the program Arlequin v. 3.5 [20]. We calculated four parameters of genetic diversity for each population: mean number of alleles *A*, observed heterozygosity *H*_o_ and expected heterozygosity *H*_e_ using the same program, while Fstat v. 2.9.3.2 [21] was used to calculate allelic richness *AR*, the mean number of alleles based on the lowest number of individuals (here 20 samples) with the rarefaction option. Further, we calculated locus- and locality-specific allele frequencies with this program.

Analyses of molecular variance (AMOVAs) were performed to partition the genetic variance on three levels: genetic variance located among populations, among individuals within populations and within individuals. Respective fixation indices were calculated with the program Arlequin. To test for potential correlations between genetic and geographical distance (isolation-by-distance), we correlated pairwise genetic distances [*F*_ST_/(1−*F*_ST_)] with the natural logarithm (ln) of the geographical distance, using the Isolation by Distance Web Service v. 3.23 (http://ibdws.sdsu.edu/) [22] with 10 000 permutations to test for significance.

### 3.6 Overall statistical analysis

We used generalized linear mixed effects models (GLMM) to test for potential relationships between the following parameters: abundance of *C. parallelus* with size of grassland site, with LUI, with the percentage of surrounding grassland (within a 500 m radius) and with the Shannon diversity index (500 m radius). In a second analysis, we tested for potential relationships between all four parameters of genetic diversity (*A*, *AR*, *H*_e_, *H*_o)_ and abundance of *C. parallelus*, land use, and all other landscape parameters. In both analyses, we included two-way interactions between land use and habitat size and landscape variables to test whether grassland size and edge-habitat-size effects depend on LUI. We used a stepwise model selection by AIC (backward and forward selection) with the function stepAIC in the package MASS in R v. 2.14.0 (http://www.r-project.org/) [23,24]. Abundance of *C. parallelus*, habitat size and percentage of surrounding grassland were ln-transformed prior to analysis to improve normality of residuals and homoscedasticity.

## 4. Results

### 4.1 Genetic diversity and differentiation

We found no significant linkage disequilibrium and deviations from Hardy–Weinberg equilibrium and only marginal effects due to null alleles. Genetic diversity was homogeneously distributed over all 22 populations, with overall means as follows: *A*=18.8±1.35 s.d., *AR*=16.5±0.8, *H*_o_=65.9%±4.7, *H*_e_=84.2%±1.9. All values are given in table 2.

**Table 2.** Parameters of genetic diversity calculated for all *Chorthippus parallelus* over all local populations and loci analysed, given for each sample site. Shown is the number of sampled individuals *N*, the mean number of alleles *A*, allelic richness *AR* based on the lowest number of sampled populations (20 individuals), percentage of observed heterozygosity *H*_o_ and percentage of expected heterozygosity *H*_e_.

site	site^*a*^	*N*	*A*	*AR*	*H*_o_ (%)	*H*_e_ (%)
1	HEG43	26	19.2	16.3	61.7	83.9
2	HEG07	22	17.2	15.4	58.8	82.1
3	HEG08	26	20.2	17.2	59.6	83.1
4	HEG10	26	19.3	16.8	74.3	84.4
5	HEG28	27	20.2	17.3	65.9	85.3
6	HEG29	26	18.7	16.6	64.7	83.9
7	HEG31	26	19.5	16.8	68.5	86.1
8	HEG05	21	17.3	16.1	62.5	81.2
9	HEG30	29	20.2	17.3	66.6	82.2
10	HEG41	30	18.8	16.5	63.7	86.5
11	HEG09	25	18.5	16.4	68.1	82.7
12	HEG06	22	16.7	15.6	71.5	87.2
13	HEG46	28	20.0	17.2	67.3	84.2
14	HEG21	24	19.3	17.7	68.8	86.3
15	HEG33	24	17.8	15.7	70.8	84.6
16	HEG04	32	19.3	15.8	61.5	85.1
17	HEG15	28	18.5	15.9	67.9	81.9
18	HEG32	29	20.8	17.6	75.3	87.4
19	HEG27	28	18.8	16.4	59.9	81.5
20	HEG16	26	18.7	16.1	62.5	82.9
21	HEG37	27	20.2	17.2	69.0	86.7
22	HEG02	20	15.2	14.8	60.9	83.8

^*a*^Name of site according to the Biodiversity Exploratories.

The genetic differentiation across all 22 local populations was very low (0.0223, *F*_ST_=0.0091, *p*>0.05). The major proportion of the genetic variance was detected among individuals within populations (0.5009, *F*_IS_=0.2058, *p*<0.0001) and within individuals (1.9336). The correlation of pairwise genetic distances [*F*_ST_/(1−*F*_ST_)] and the natural logarithm of the geographical distances over our 40 km×40 km study range showed no significant relationship (Mantel test: *p*=0.602; 10 000 permutations). Genetic and geographical pairwise distances are given in the electronic supplementary material, S1 and S2; all molecular data generated for this study are provided as genepop inputfile in the electronic supplementary material, S3.

### 4.2 Effects of land use and landscape

Habitat size, LUI and surrounding landscapes differed among the studied sites. Habitat size ranged from 2.63 to 128 ha (with a mean of 21.4±29.3 ha s.d.). LUI varied from very low (0.59) to very high (2.73) (with a mean of 1.67±0.67 s.d.). The percentage of grassland in the adjoining environment varied from 0 to 88.0% (with a mean of 39.4±32.2%). The heterogeneity of the surrounding environment measured as Shannon diversity index ranged from 0.00 to 1.12 (0.62±0.29). Values for each site are given in table 1.

**Figure 2.**
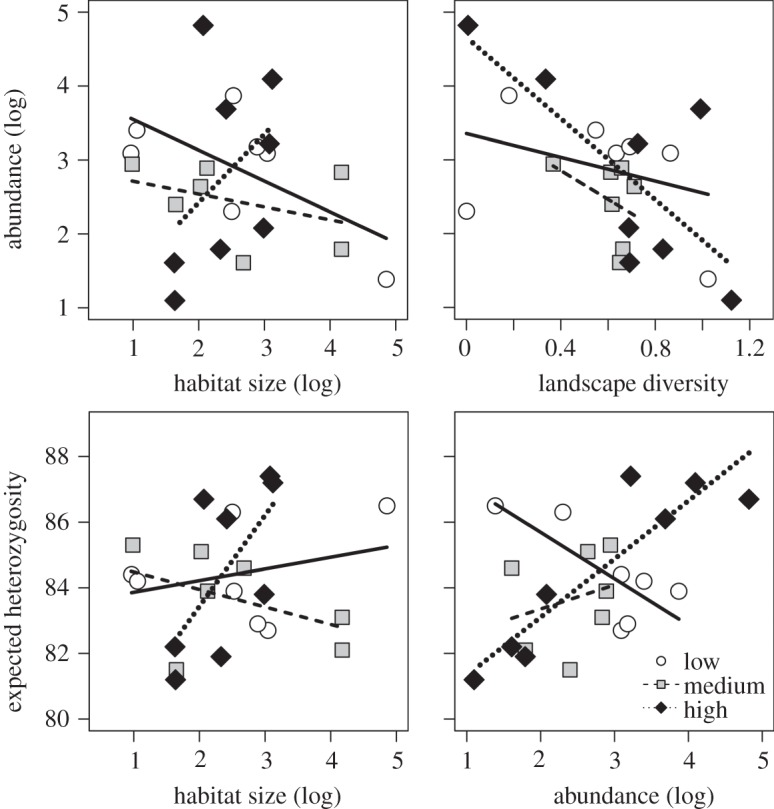
Abundance of *Chorthippus parallelus* as a function of habitat size and landscape diversity and expected heterozygosity as a function of habitat size and the abundance of *C. parallelus*.

The sweep netting resulted in total numbers of *C. parallelus* individuals between 3 and 124 per site (mean 24±2.97 s.d.). The abundance of the grasshopper species was significantly correlated with the size of patches, habitat heterogeneity of the surrounding environment and LUI (figure 2 and table 3). The abundance was negatively correlated with habitat size at low and medium LUI; a positive relationship was observed at high LUI. Landscape heterogeneity generally affected the abundance of *C. parallelus* negatively, but the relationship was stronger at medium and high LUI.

**Table 3.** Results of generalized linear mixed effects models (GLMM) on the relationship between abundance and genetic diversity (mean number of alleles *A*, percentage of observed heterozygosity *H*_o_, expected heterozygosity *H*_e_, allelic richness *AR*), habitat size, LUI and landscape variables in the surrounding buffer (radius=500 m). Final models were defined by stepwise model selection using AIC (backward and forward). Grey shaded cells indicate variables that are deleted during model simplification.

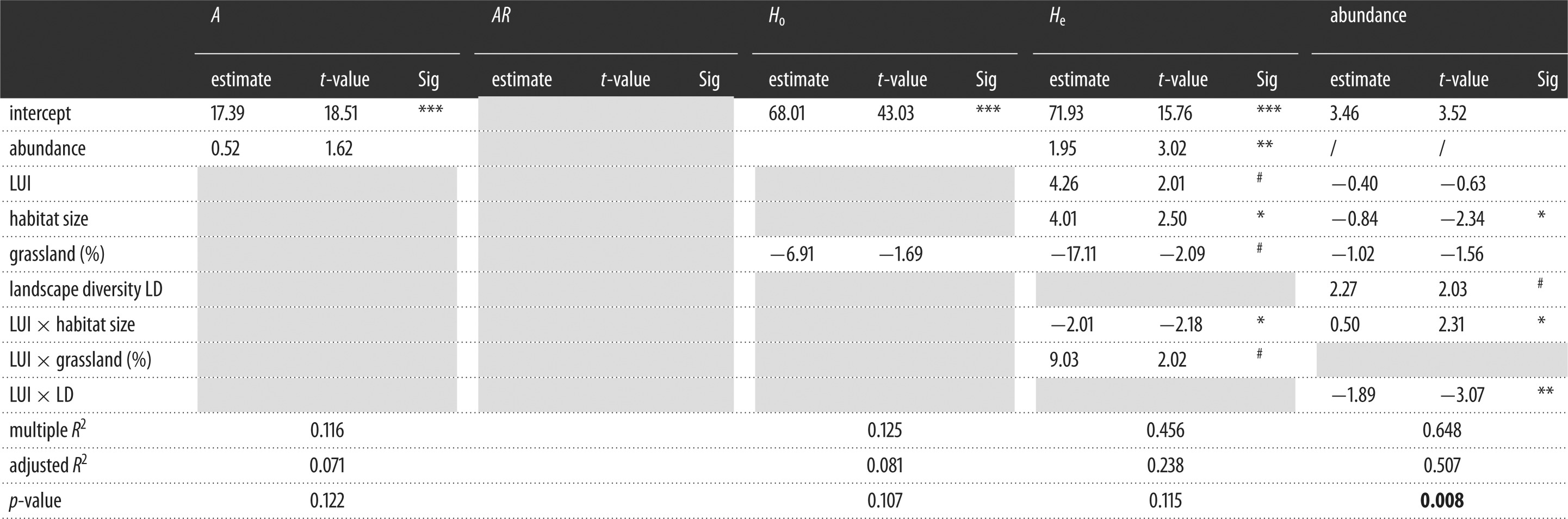

***p<0.001, **p<0.01, *p<0.05, ^#^*p*<0.10.

Genetic diversity was not strongly affected by habitat size, LUI and the surrounding landscape, except for the expected heterozygosity. Here, a positive relationship between habitat size and *H*_e_ could be observed at high LUI (figure 2). Further, *H*_e_ was negatively affected by the abundance at low LUI, but positively at medium and high LUI (figure 2).

## 5. Discussion

### 5.1 Genetics of a widespread and abundant invertebrate

The genetic analysis on the 22 local populations of the common and abundant meadow grasshopper revealed a low level of genetic differentiation across all populations, no isolation-by-distance, and an almost homogeneously distributed genetic diversity. Correlations between abundance, parameters of genetic diversity and biotic and abiotic characteristics of the grassland sites were significant yet only weak. These results suggest that the strong fragmentation of grassland habitats has no effect on the genetic diversity and differentiation of *C. parallelus.*Negative effects of fragmentation such as genetic impoverishment and strong population differentiation often observed in organisms living in similar fragmented habitats may be overcome by large population sizes and strong gene flow among local sub-populations.

Our data are in line with the high abundance of *C. parallelus*, but contradict the general assumption that this grasshopper species is mostly flightless and shows rather low dispersal ability [9,12]. The species' comparatively broad ecological tolerance allows the species to inhabit a variety of habitat types within a large geographical range and leads to its widespread occurrences and high abundances [12]. The observed low genetic differentiation and homogeneously distributed and high genetic diversity have frequently been observed for many generalist grasshopper species, while, in contrast, specialist species often show opposite genetic patterns indicating low genetic diversity and strong differentiation [25–28]. Similar trends can be found for other invertebrates, such as butterflies: here the most specialized butterflies of Europe, representatives of the genus *Maculinea*, show a comparatively low genetic diversity and high differentiation among local populations, while other lycaenids such as the widespread *Polyommatus icarus* or *Polyommatus coridon* show a comparatively high genetic diversity and low genetic differentiation [4].

### 5.2 Land-use intensity, species abundance and genetic diversity

The abundance of *C. parallelus* was not significantly affected by LUI. This opposes other ecological studies documenting a reduction in Orthopteran abundance due to the impact of mowing [29,30]. Humbert *et al*. [29,30] and Gardiner & Hassall [31] suggested that lowered abundances might be the result of a combination of mortality caused directly by the physical damage during mowing as well as the high sward temperatures created by removal of the standing crop. In their study, the abundance of *C. parallelus* and *Chorthippus albomarginatus* showed a significant decline in the abundance in study plots which are mowed compared with unmanaged control swards (which we did not incorporate in our analysis) [31]. Generally, there is an impact continuum of grassland management on grasshopper populations, depending mainly on date of the season, and exact method and frequency of management [11], so that LUI will somewhat superpose the different local situations.

In our study, the abundance of *C. parallelus* was significantly affected by habitat size and the heterogeneity of the surrounding environment, yet the direction and significance of the respective relationship depended on LUI. *Chortippus parallelus* seems to benefit from increasing habitat size but suffers from higher environmental heterogeneity under high LUIs. Greater patch sizes and lower landscape diversity in the surrounding patches probably result in larger areas of suitable habitat and subsequent larger population sizes and increasing connectivity.

The strong variation in the abundances of *C. parallelus* across our study area might additionally be the result of differences in abiotic conditions such as microclimate (temperature, humidity), or different plant species compositions [11]. Previous studies showed that the fecundity of *C. parallelus* is positively affected by the temperature and moisture of the grasslands [32], and that diet can strongly affect its fitness [8,33]. Thus, differences among local population abundances might rather come from different biotic and abiotic factors such as soil condition (humidity), elevation and inclination—having a higher relevance than the LUIs. A final factor affecting the values of the abundance of the grasshopper might be due to sampling bias.

Taking into account that with 10 double sweeps about 8 m^2^ are swept, and that only 25% of all individuals become sampled [11], our results from sweep-netting underline that the local population sizes must be very high, ranging from several thousands to some tens of thousands per study site (50 m×50 m). The high and equally distributed genetic diversity found within populations might be the result of high population sizes ranging at a very high level—at which differences might play a rather negligible role for potential genetic effects [34].

Genetic parameters showed no significant correlation with any of the habitat characteristics (e.g. size of the grassland site, LUI, the percentage of grassland in adjoining landscape or heterogeneity of the surrounding environment). Only the expected heterozygosity was significantly affected by the abundance of *C. parallelus* and by habitat size; this correlation was strongly positive but only found in plots with high LUI. Furthermore, this goes in line with higher abundances of *C. parallelus* found in these plots. This relationship between population sizes and genetic diversity (here expressed by expected heterozygosity) can frequently be observed in wild populations [35]. However, expected heterozygosity is an estimate (and not measurements of *in situ* diversity found in individuals) based on allele frequencies and the Hardy–Weinberg equilibrium assumptions, which makes the significance of this correlation between LUI and genetic diversity arguable [36].

In conclusion, the lack of effects from different LUIs can be explained by two scenarios. First, the unspecific habitat requirements and high ecological tolerance of *C. parallelus* may lead to high abundances, even in landscapes with intensive land-use regimes. This allows the species to exist in large population sizes counteracting potential genetic drift effects. It needs to be added that the total area of grassland in the study area is still quite large, so possibly stronger fragmentation and decreased total habitat area may lead to a decrease in genetic diversity and higher differentiation. Second, even if habitats and populations are small (as observed in some sites for some years), high abundances can easily counteract potential drift effects by gene flow from adjoining populations, and thus prevent the loss of genetic diversity. These two explanations might play a pivotal role in buffering potential effects of genetic drift, and finally in producing a population genetic structure characterized by high diversity and low differentiation.

## Data accessibility

Allelic data are uploaded as Supplementary Material.

## References

[1] Fahrig L 2003 Effects of habitat fragmentation on biodiversity. *Annu. Rev. Ecol. Evol. Syst.* 34, 487–515. (doi:10.1146/annurev.ecolsys.34.011802.132419)

[2] Melbourne BA, Hastings A 2008 Extinction risk depends strongly on factors contributing to stochasticity. *Nature* 454, 100–103. (doi:10.1038/nature06922) 18596809 10.1038/nature06922

[3] Shifman S, Darvasi A 2001 The value of isolated populations. *Nat. Genet.* 28, 309–310. (doi:10.1038/91060) 11479587 10.1038/91060

[4] Habel JC, Schmitt T 2012 The burden of genetic diversity. *Biol. Conserv.* 147, 270–274. (doi:10.1016/j.biocon.2011.11.028)

[5] Keller I, Nentwig W, Largiader CR 2004 Recent habitat fragmentation due to roads can lead to significant genetic differentiation in an abundant flightless ground beetle. *Mol. Ecol.* 13, 2983–2994. (doi:10.1111/j.1365-294X.2004.02310.x) 15367114 10.1111/j.1365-294X.2004.02310.x

[6] Dennis RLH, Eales HT 1997 Patch occupancy in Coenonympha tullia (Muller, 1764) (Lepidoptera: Satyrinae): habitat quality matters as much as patch size and isolation. *J. Insect Conserv.* 3, 167–176. (doi:10.1023/A:1018455714879)

[7] Vergeer P, Rengelink R, Copal A, Ouborg NJ 2003 The interacting effects of genetic variation, habitat quality and population size on performance of Succisa pratensis. *J. Ecol.* 91, 18–26. (doi:10.1046/j.1365-2745.2003.00736.x)

[8] Franzke A, Unsicker SB, Specht J, Köhler G, Weisser WW 2010 Being a generalist herbivore in a diverse world: how do diets from different grasslands influence food plant selection and fitness of the grasshopper Chorthippus parallelus. *Ecol. Entomol.* 35, 126–138. (doi:10.1111/j.1365-2311.2009.01168.x)

[9] Unsicker SB, Franzke A, Specht J, Köhler G, Linz J, Renker C, Stein C, Weisser WW 2010 Plant species richness in montane grasslands affects the fitness of a generalist grasshopper species. *Ecology* 91, 1083–1091. (doi:10.1890/09-0402.1) 20462122 10.1890/09-0402.1

[10] Specht J, Scherber CH, Unsicker SB, Köhler G, Weisser WW 2008 Diversity and beyond: plant functional identity determines herbivore performance. *J. Anim. Ecol.* 77, 1047–1055. (doi:10.1111/j.1365-2656.2008.01395.x) 18844757 10.1111/j.1365-2656.2008.01395.x

[11] Ingrisch S, Köhler G 1998 *Die Heuschrecken Mitteleuropas (Die Neue Brehm-Bücherei).* Magdeburg, Germany: Westarp Wissenschaften.

[12] Fischer M etal 2010 Implementing large-scale and long-term functional biodiversity research: the biodiversity exploratories. *Basic Appl. Ecol.* 11, 473–485. (doi:10.1016/j.baae.2010.07.009)

[13] Blüthgen N etal 2012 A quantitative index of land-use intensity in grasslands: integrating mowing, grazing and fertilization. *Basic Appl. Ecol.* 13, 207–220. (doi:10.1016/j.baae.2012.04.001)

[14] Köhler G, Perner J, Schumacher J 1999 Grasshopper population dynamics and meteorological parameters—lessons from a case study. *Ecography* 22, 205–212. (doi:10.1111/j.1600-0587.1999.tb00469.x)

[15] Wiesner KR, Loxdale HD, Köhler G, Schneider ARR, Tiedemann R, Weisser WW 2011 Patterns of local and regional genetic structuring in the meadow grasshopper, Chorthippus parallelus (Orthoptera: Acrididae), in Central Germany revealed using microsatellite markers. *Biol. J. Linn. Soc.* 103, 875–890. (doi:10.1111/j.1095-8312.2011.01698.x)

[16] Abercrombie etal 2009 Primer note—permanent genetic resources added to Molecular Ecology Resources database 1 January 2009–30 April 2009. *Mol. Ecol. Resources* 9, 1375–1379. (doi:10.1111/j.1755-0998.2009.02746.x) 10.1111/j.1755-0998.2009.02746.x 21564911

[17] Miller SA, Dykes DD, Polesky HF 1988 A simple salting out procedure for extracting DNA from human nucleated cells. *Nucleic Acids Res.* 16, 1215 (doi:10.1093/nar/16.3.1215) 3344216 10.1093/nar/16.3.1215 PMC334765

[18] Pfautsch S, Schneider ARR, Wiesner KR, Weisser WW, Tiedemann R 2010 Nine new microsatellite markers for the meadow grasshopper (Chorthippus parallelus). *Mol. Ecol. Res.* 9, 1375–1379.

[19] Van Oosterhout C, Hutchinson WF, Wills DPM, Shipley P 2004 MICRO-CHECKER: software for identifying and correcting genotyping errors in microsatellite data. *Mol. Ecol. Notes* 4, 535–538. (doi:10.1111/j.1471-8286.2004.00684.x)

[20] Excoffier L, Laval G, Schneider S 2005 ARLEQUIN (version 3.0): An integrated software package for population genetics data analysis. *Evol. Bioinform.* 10, 47–50. PMC2658868 19325852

[21] Goudet J 1995 FSTAT (Version 1.2): a computer program to calculate F-statistics. *J. Heredity* 86, 485–486.

[22] Jensen JL, Bohonak AJ, Kelley ST 2005 Isolation by distance, web service. *BMC Genetics* 6, 13 (doi:10.1186/1471-2156-6-13) 15760479 10.1186/1471-2156-6-13 PMC1079815

[23] Venables WN, Ripley BD 2002 *Modern applied statistics with S* 4th edn. New York, NY: Springer.

[24] R Core Team. 2010 R: A language and environment for statistical computing. (http://www.R-project.org/)

[25] Habel JC, Rödder D, Lens L, Schmitt T 2013 The genetic signature of ecologically diverging grassland lepidopterans. *Biodivers. Conserv.* 22, 2401–2411. (doi:10.1007/s10531-012-0407-y)

[26] Ortego J, Aguirre MP, Cordero PJ 2010 Population genetics of Mioscirtus wagneri, a grasshopper showing a highly fragmented distribution. *Mol. Ecol.* 19, 472–483. (doi:10.1111/j.1365-294X.2009.04512.x) 20051009 10.1111/j.1365-294X.2009.04512.x

[27] Ortego J, Aguirre MP, Cordero PJ 2012 Landscape genetics of a specialized grasshopper inhabiting highly fragmented habitats: a role for spatial scale. *Divers. Distrib.* 18, 481–492. (doi:10.1111/j.1472-4642.2011.00840.x)

[28] Ortego J, Bonal R, Cordero PJ, Aparicio MG 2009 Phylogeography of the Iberian populations of Mioscirtus wagneri (Orthoptera: Acrididae), a specialized grasshopper inhabiting highly fragmented hypersaline environments. *Biol. J. Linn. Soc.* 97, 623–633. (doi:10.1111/j.1095-8312.2009.01206.x)

[29] Humbert JY, Ghazoul J, Richner N, Walter T 2010 Hay harvesting causes high orthopteran mortality. *Agric. Ecosyst. Environ.* 44, 354–355.

[30] Humbert JY, Ghazoul J, Sauter GJ, Walter T 2010 Impact of different meadow mowing techniques on field invertebrates. *J. Appl. Entomol.* 134, 592–599.

[31] Gardiner T, Hassall M 2009 Does microclimate affect grasshopper populations after cutting of hay in improved grassland? *J. Insect Conserv.* 13, 97–102. (doi:10.1007/s10841-007-9129-y)

[32] Ingrisch S 1983 Zum Einfluß der Feuchte auf die Schlupfrate und Entwicklungsdauer der Eier mitteleuropäischer Feldheuschrecken (Orthoptera, Acrididae). *Deutsche Entomol. Zeitschrift* 30, 1–15. (doi:10.1002/mmnd.19830300102)

[33] Unsicker SB, Oswald A, Köhler G, Weisser WW 2008 Complementarity effects through dietary mixing enhance the performance of a generalist insect herbivore. *Oecologia* 156, 313–324. (doi:10.1007/s00442-008-0973-6) 18301924 10.1007/s00442-008-0973-6 PMC2757592

[34] Slatkin M 1987 Gene flow and the geographic structure of natural populations. *Science* 236, 787–792. (doi:10.1126/science.3576198) 3576198 10.1126/science.3576198

[35] Frankham R 2002 Relationship of genetic variation to population size in wildlife. *Conserv. Biol.* 10, 1500–1508. (doi:10.1046/j.1523-1739.1996.10061500.x)

[36] Nei M 1978 Estimation of average heterozygostiy and genetic distance from a small number of individuals. *Genetics* 89, 3583–3590. 10.1093/genetics/89.3.583 PMC1213855 17248844

